# Influence of different size of prophylaxis brush on marginal microleakage and hybrid layer morphology of composite restorations

**DOI:** 10.4317/jced.62831

**Published:** 2025-07-01

**Authors:** Monique Evelin Pereira Da Silva, Maria Eduarda Da Silva Mendes, Tayná Souza Gomes da Silva, Danielle Ferreira Sobral-Souza, Marlon Ferreira Dias, Paulo Cardoso Lins Filho, Hilcia Mezzalira Teixeira, Audrey Nunes de Andrade, Antonio José Torres Neto, Renata Pedrosa Guimarães

**Affiliations:** 1 Graduated in Dentistry at Federal University of Pernambuco (UFPE), Department of Prosthesis and Oral and Facial Surgery, Recife, Pernambuco, Brazil; 2 Professor of Dentistry at Federal University of Mato Grosso do Sul (UFMS), School of Dentistry, Campo Grande, Mato Grosso do Sul, Brazil; 3 PhD student at São Paulo State University (UNESP), School of Dentistry, Department of Dental Materials and Prosthodontics, Araraquara, São Paulo, Brazil; 4 Researcher at the Center for Strategic Technologies of the Northeast, Brazil; 5 Professor of Dentistry at Federal University of Pernambuco (UFPE), Department of Prosthesis and Oral and Facial Surgery, Recife, Pernambuco, Brazil

## Abstract

**Background:**

Tooth cavity cleaning is a fundamental step to achieve greater adhesion in composite resins restorations. This study aimed to evaluate the influence of different sizes of prophylaxis brush on marginal microleakage and hybrid layer morphology in composite restorations.

**Material and Methods:**

Thirty Class V tooth preparations were distributed into 3 groups according to the type of cavity cleaning (n=10): G1: no prophylaxis; G2: Prophylaxis brush with regular size (Robson Brush Prophylaxis/DHPro); G3: Prophylaxis brush with reduced size (Robson Microtuft®/DHPro brush). After the respective cleaning protocol, third human molars were restored using a universal adhesive system (Single Bond Universal - 3M ESPE) and composite resin (FiltekTM Z350 XT 3M ESPE). To characterize the dentin substrate, the samples were prepared until the selective conditioning step and evaluated by scanning electron microscope (SEM) and hybrid layer’s morphology was also evaluated after the restorative procedure (n=2). To assess the marginal microleakage, the sample were submitted to the thermocycling process, finished, and polished. After this step, the images were obtained by Micro-CT, and two calibrated evaluators scored the depth of dye penetration as 0 to 3. The Kruskal-Wallis test was used to compare groups regarding marginal microleakage (*p*<0.05).

**Results:**

G1 and G2 showed the presence of a gap between restoration and dentin, however, G3 showed greater regularity compared to the other groups. In microleakage scores, G1 showed more frequency for score 3 followed by G3 e G2 (*p*>0.05).

**Conclusions:**

Therefore, the Robson Microtuft® brush did not influence the marginal infiltration of the adhesive restorations, but the absence of prior prophylaxis increased the levels of infiltration and obtained a more irregular adhesive interface.

** Key words:**Microleakage. marginal adaptation. universal adhesives.

## Introduction

The popularity of composite restorations is due to the development of adhesive systems, which allows more conservative tooth preparations and composite resins’ aesthetic characteristics (1,2). However, some factors, such as differences in chemical formulations, physical properties, and insertion techniques, can influence the longevity of composite restorations (3-5).

Universal adhesives system simplified the restorative procedure with the one-step application (6,7). However, these adhesives have lower conditioning capacity and partially dissolve the smear layer, which can affect the final result of the adhesive interface (8,9). In tooth preparations, the cleaning step assumes a fundamental step in adhesive procedures, providing effective cleaning of the dentin substrate, increasing surface energy, and facilitating the wetting of the adhesive in dentin (10). However, in dental practice dealing with deeper cavities, the prophylaxis step using a regular-size brush for cleaning may not be efficient due to their larger diameter.

The prophylaxis brushes with reduced size are a new option to clean deep tooth preparations, resulting in better cleaning and possibly improving adhesion. This assumes greater importance also in areas that are more critical for good adhesion, such as the cervical region in non-carious lesions, and during aesthetic procedures such as direct veneers and cementation of ceramic laminates.

Thus, the objective of this study was to evaluate the morphology of the hybrid layer by scanning electron microscopy (SEM) and marginal microleakage in class V composite resin restorations using different sizes of prophylaxis brushes. The null hypothesis tested for this study was that using a reduced size of prophylaxis brush did not interfere hybrid layer’s morphology and marginal microleakage.

## Material and Methods

- Specimens obtaining 

In this *in vitro* study, 15 human third molars with more than 2/3 of the root portion formed were included. Teeth extracted more than six months ago or had enamel cracks, restorations, carious lesions, noncarious cervical lesions, or developmental lesions visible to the naked eye were not included. Afterward, the teeth were cleaned with a McCall universal periodontal curette (Millennium Golgran, São Caetano do Sul, SP, Brazil), and a prophylaxis brush using a pumice/water paste was performed. The samples were immersed in a 2% chlorhexidine solution (Riohex, São José do Rio Preto, São Paulo, Brazil) for 12 hours to disinfection. Then, the samples were stored in a 0.9% sodium chloride solution (changed weekly) and kept in a refrigerator.

- Preparation of Samples

All teeth (n=15) received two cervical preparations (buccal and lingual), measuring approximately 5 mm in width, 2 mm in height, and 2 mm in depth, resulting in 30 class V preparations. A hollow plastic mold was used to create the cavity preparation. This plastic mold was measured with a millimeter ruler and cut with the aid of a scalpel blade. This mold was placed on the tooth to mark the cavity contour, and then with a millimeter probe (Millennium Golgran, São Caetano do Sul, SP, Brazil), the measurements were checked. The cavity preparations were made under high rotation and refrigerated conditions with FG 1014 spherical diamond tips (KG Sorensen, São Paulo, Brazil).

- Experimental Procedure

The samples were randomly distributed (n=10), according to the type of previous treatment performed: G1: no prophylaxis (negative control), G2: prophylaxis brush of regular size for cleaning (Robson brush, positive control) G3: prophylaxis brush of reduced size for cleaning (Robson brush Microtuf®, DHPro). The prophylaxis technique applied in this study used pumice stone and water for 20 seconds due to dental clinic practicality.

The sample was restored using a universal adhesive system (Single Bond Universal - 3M ESPE, Saint Paul, MN, USA), according to the manufacturer’s guidelines for the selective enamel conditioning technique, 37% phosphoric acid (Condac 37 - FGM, Joinville, SC, Brazil) and composite resin (Filtek™ Z350 XT 3M ESPE, Saint Paul, MN, USA). The light-curing was carried out with an LED Emitter C device (Schuster, Santa Maria, RS, Brazil), whose intensity (approx. 900mW/cm²) was measured by a radiometer device (Demetron®) before each work step. Teeth that did not receive restoration were prepared until the selective etching step with phosphoric acid, to characterize the dentin substrate using SEM (n=2). The description of the adhesive protocol used is described in  Table 1.

- Marginal Microleakage 

After the restorative procedure, the teeth were subjected to 125 thermocycling between 5 ± 5°C and 55 ± 5°C., with 15 seconds in each bath and 3 to 5 seconds of transfer time between baths. After 5 days, the restorations were subjected to the finishing process and final polishing.

At the end of this process, the teeth surfaces were covered with two coats of nail varnish up to 1 mm before the restoration limits. The samples were immersed in a silver nitrate solution (AgNO3, 50% w/m) for 24 hours. Concentrated ammonium hydroxide (28%) was used to titrate the dark solution until it became clear as the carbon ions ammonia to convert silver into silver diamine ions. After that, the images were obtained using a Micro-CT machine (XT H 225 ST, Nikon Metrology Inc., Brighton, MI, USA).

For each restoration, a video was created in the ImageJ software to scan the entire perimeter of the restoration. A total of 5 videos per group were produced and sent to two evaluators, experts in the field, blind to the methodology, previously calibrated, which assigned each restaurant a score from 0 to 3, according to the following infiltration levels: Grade 0, when there was no dye explosion in the interface between the tooth and the restoration; Grade 1, when the discovery of the dye occurred up to half or so of the surrounding wall; Grade 2 if the darkness of the dye located in more than half of the surrounding wall; Grade 3 when the dye was seen reaching the axial wall (11).

- Scanning Electron Microscopy (SEM)

The samples were cut with a diamond disk (KG Sorensen) attached to a handpiece for analysis by SEM. The restored samples were submitted to a sagittal cut and polished with the two larger-grained praxis sandpaper (TDV) discs, while those that received only the preparation were submitted to a longitudinal cut. All the samples were stored in a 0.9% sodium chloride solution until the preparation for analysis.

For the analysis of the adhesive interface in SEM, the samples restored with composite resin were prepared according to a previous study (12).

## Results

**Figure 1:**
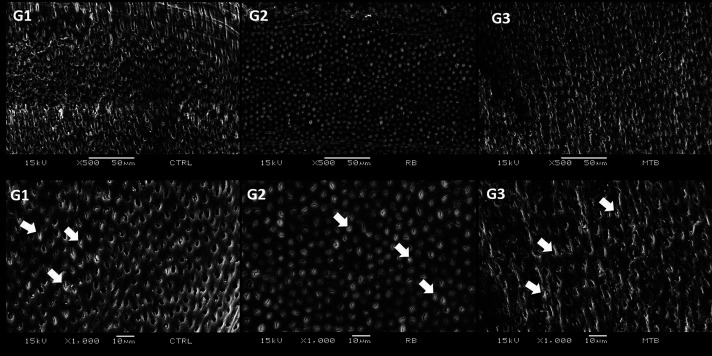
Electron micrographs representing characterization of the dentin substrate of experimentals and control group (SEM, original magnification of 500x and 1000x). G1 (no prophylaxis, negative control), G2: Prophylaxis brush with regular size: Note the presence of smear layer on the dentin surface and inside the dentinal tubules (smear plugs-white arrows); G3: Prophylaxis brush with reduced size: Note the irregular presence of smear plug (white arrows) and an appearance of an abraded surface, possibly due to the action of the prophylaxis brush (Microtuft, DHPro).

**Figure 2:**
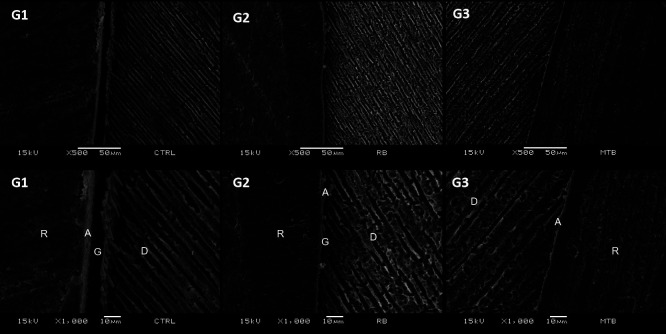
Electron micrographs represent adhesive interfaces after experimentals and control group (SEM, original magnification of 500x and 1000x). G1 (no prophylaxis, negative control), G2 (Prophylaxis brush with regular size): Presence of a gap (G) between the dentin surface (D) and the restoration (R). Adhesive(A). G3 (Prophylaxis brush with reduced size): The presence of a gap between the dentin surface (D) and the restoration (R) is not observed Adhesive (A).

Figure 1 shows a more irregular surface in G3 (Prophylaxis brush with reduced size) compared to other groups. However, this group showed greater regularity in the interface between restorative material and dentin in comparison to G1 and G2 (Fig. 2). The presence of a gap between restoration and dentin was observed in G1 and G2 (Fig. 2). Among all the groups, G1 presented a higher frequency of microleakge, score 3, followed by G3 and G2. Although, there was no statistical difference in marginal microleakage in all groups tested (*p*>0.05;  Table 2).

## Discussion

The clinical success of composite resin restorations is necessary to maintain the adhesive interface longevity (13). When there is efficient adhesion between the restorative material and the dental substrate, in addition to greater retention of the restoration, most secondary carious lesions resulting from marginal microleakage are avoided, as well as marginal staining and postoperative sensitivity (3).

The evolution of adhesive systems resulted in materials and protocols that provide a long-term adhesive bond and technique simplification (12). Considering that total acid etching has limitations, such as the possibility of causing excessive dentin conditioning, which results in poor adhesion, self-etching adhesive systems emerged with the attraction of making the technique less sensitive. These systems also simplify the restorative technique by reducing the number of steps by dispensing acid conditioning, consequently reducing the clinical time of the procedure (13,14).

The universal adhesive system has a formulation similar to the self-etching adhesive system. In the composition of these materials, functional monomers that chemically bind to the calcium of hydroxyapatite improve adhesion, such as 10-MDP present in the composition of universal adhesives (13,14). Although this material can be used in total conditioning, self-conditioning, and selective conditioning modes (13), in the present study selective conditioning was performed.

The findings of this study reinforce the critical role of surface treatment protocols in optimizing the bond strength between composite resin and dental tissues. The use of pumice and water—a common prophylactic method (15) in clinical settings—showed the lowest bond strength. Its function is primarily to remove the acquired pellicle and biofilm, but it does not modify the enamel or dentin structure in a way that favors adhesion. While it serves as a cleaning agent, our findings confirm its inadequacy as a surface treatment for adhesive procedures. Nevertheless, some studies (3,16) suggest that prophylaxis with pumice may increase the surface energy of the substrate, potentially benefiting adhesive wetting when associated with further surface treatments. Surface energy is crucial to achieving the success of the adhesive interface, and prophylaxis with pumice is one of the ways to increase the surface energy of cavity preparation in restorative procedures (3,16). Therefore, the use of compatible-size toothbrushes in the prophylaxis stage of the restorative procedure is crucial to ensure a higher-quality adhesive interface and, consequently, a satisfactory restoration result.

In this study, the qualitative analysis of the hybrid layer was performed by Scanning Electron Microscopy (SEM). Several researchers (17,18) widely use this method as it is the best known, most accurate, and simple tool to assess the morphology of the adhesive/dentin interfaces and the quality of the hybrid layer.

SEM analysis revealed different substrates for each treatment performed on specimens that did not receive the restoration. In G1, the images showed a surface with a smear layer and smear plugs in the dentinal tubules. This is because the cavity preparation causes an accumulation of organic and inorganic residues that form a smear layer. This layer also fills the holes of the dentinal tubules forming the so-called smear plugs (19). However, in the images, it is possible to observe some unobstructed tubules, which can be explained by the preparation method for evaluation in SEM since these samples were submitted to ultrasound.

In the images obtained in G2, a more uniform smear layer is observed, in addition to the presence of obstructed tubules while in G3, an irregular smear layer was observed, which may have been caused by friction of the brush bristles in contact with the surface. This result corroborates with a previous study (20), although the analysis was carried out to biofilm analysis. The study investigated the effectiveness of various biofilm removal techniques and whether residual biofilm on the dentin surface would affect the adhesion process. It was concluded that prophylaxis with a rubber cup and pumice stone removed the biofilm to a significant degree. However, some bacterial cells still partially covered the dentin surface and obstructed the dentinal tubules. It should be considered that the study mentioned above evaluated the effectiveness of free surface prophylaxis. In small-sized cavities as well as in difficult-to-access areas, such as cervical regions close to the gingival tissue, the use of abrasive brushes with a compatible size can significantly contribute to the effectiveness of prophylaxis and consequently to the quality of adhesive procedures as seen in the images obtained in this study (Figs. 1,2).

SEM analyses revealed adhesive interfaces with evident differences in samples that received the restoration. In group G1, an extensive gap between dentin and restorative material was observed. The gap represents a space between the restorative material and the tooth structure and may have the clinical consequences of marginal discoloration of the restoration, secondary caries, and postoperative sensitivity (21). A gap between the restorative material and the dentin was observed in G2. However, compared to the G1 group, the extent of this gap was smaller (Fig. 2).

The presence of gaps in G1 and G2 can be explained because there was no efficiency in cleaning the cavity. In the use of self-etching adhesive systems, it is possible that thicker smear layers can prevent the correct infiltration of the acid monomer and, consequently, compromise the bond strength by not forming a true hybrid layer. At the time of application, when acidic monomers try to infiltrate the smear layer, the minerals present in this layer buffer the monomers, thus, infiltration is hampered by the gradual loss of acidity and the self-infiltration capacity of these substances (8,22,23). The infiltration of monomers in the smear layer is of fundamental importance for forming chemical bonds with the tooth surface (9).

However, the SEM images obtained in G3 (prophylaxis brush with reduced size) revealed adhesion without gaps, showing a better interaction between the adhesive interfaces. This result also corroborates a previous study (20), which concluded that the adhesion of dentin covered by the biofilm was improved after mechanical removal of the biofilm through prophylaxis with a pumice stone. Niyomsujarit, Senawongse, and Harnirattisai (2019) (24), associated brushing and ultrasonic cleaning for 30 seconds before applying the adhesive, there was also an improvement in bond strength after this combination. The preliminary findings of the present study encourage the performance of a mechanical micro tensile test, according to the same protocols used in this study, to corroborate the clinical relevance of the results obtained.

Several materials are used to clean the cavity and improve the resin-dentin bond, such as pumice stone, chlorhexidine, aluminum oxide, and sodium hypochlorite (18,25-27). Frassetto *et al*. (2015) (28) also cite ethanol, while a study by Bandeira *et al*. (2019) (29) also cites an emulsion of copaiba oil (CO), an exudate secreted from the trunk of copaiba trees, as a cleaning agent for dentin. In this study, only a pumice stone was used to perform the prophylaxis step, which is a limitation of this research. Furthermore, quantitative tests must also be performed to confirm the success of adhesion in restorative treatment.

The performance of the universal adhesive system used in this study—applied with the selective enamel etching technique—demonstrated promising results. These systems, which contain functional monomers like 10-MDP, promote chemical interaction with hydroxyapatite and allow for versatile application modes (self-etch, total-etch, or selective-etch). Selective etching with phosphoric acid followed by the universal adhesive provided effective bonding, particularly in groups where prophylaxis was optimized.

Surface energy plays a crucial role in the quality of the adhesive interface (30). SEM analysis showed that when no adequate cleaning or etching was performed (G1), the dentin surface remained covered by a smear layer and smear plugs, leading to evident interfacial gaps. These defects can contribute to clinical complications such as marginal discoloration, secondary caries, and postoperative sensitivity (1). Conversely, samples treated with reduced-size prophylactic brushes (G3) revealed cleaner surfaces and more homogeneous adhesive interfaces, with no detectable gaps. These findings align with previous literature emphasizing the benefits of mechanical cleaning for improving adhesion (24).

Despite the observed differences in interfacial quality, marginal microleakage analysis revealed no statistically significant differences among the groups. However, qualitative observations from SEM imaging suggest that prophylactic protocols using appropriately sized brushes may contribute to a more regular interface and reduce clinical failure risks.

The images showed that not removing the smear layer decreases the dentin’s surface energy and negatively affects the adhesive’s wettability, resulting in poor adhesion. With this information and knowledge of the different situations that can increase the substrate’s surface energy, the dentist should use techniques that aim to improve adhesion (3).

This study has certain limitations that must be acknowledged. The *in vitro* nature of the experimental design does not fully replicate the complex conditions of the oral environment, such as saliva contamination, temperature fluctuations, and masticatory forces, which may influence adhesive behavior. Moreover, the study focused exclusively on immediate bond strength, without evaluating long-term durability or the effects of aging processes such as thermocycling or water storage. Despite these constraints, the findings provide valuable insights into the efficacy of different surface treatments and their potential to improve clinical outcomes. As adhesive dentistry continues to be a cornerstone of minimally invasive restorative procedures, understanding which pre-treatment methods yield superior enamel-resin interfaces is vital for ensuring long-term success and reducing clinical failures. Our data contribute to this body of knowledge, emphasizing the importance of selecting evidence-based protocols tailored to each restorative indication.

## Conclusions

Within the limitations of this *in vitro* study, it can be concluded that the use of 37% phosphoric acid for 30 seconds remains the most effective enamel surface treatment for improving the bond strength of composite resins. While pumice and water alone do not enhance bond strength, their use in conjunction with appropriate prophylactic brushes showed improved surface cleanliness and a more regular adhesive interface under SEM analysis. These findings underscore the importance of selecting appropriate surface treatments not only for mechanical retention but also for optimizing the quality of the adhesive interface. Incorporating evidence-based surface conditioning protocols remains fundamental for achieving predictable and durable outcomes in adhesive restorative procedures.

## Figures and Tables

**Table 1:** Description of the adhesive protocol used in this study.

Groups	Selective acid conditioning technique in enamel
G1	1. Phosphoric acid for 15 seconds 2. Wash well with water 3. Dry with cotton balls 4. Actively apply the adhesive for approximately 20 seconds 5. Air blast for 5 seconds 6. Light cure for 10 seconds 7. Insert the material restorer
G2	
G3	

G1: no prophylaxis (control); G2: Prophylaxis brush with regular size (Robson Brush Prophylaxis/DHPro); G3: Prophylaxis brush with reduced size (Robson Microtuft®/DHPro brush).

**Table 2:** Marginal microfltration score, median and interquatil ranges according to each goup.

Groups	Score 0		Score 1		Score 2		Score 3		Total		Median (IQR*/IQR**)	p value¹
	f	%	f	%	f	%	f	%	n	%		
G1	0	0%	1	10%	0	0%	9	90%	10	100%	3,00 (3,00/3,00)	
G2	1	10%	2	20%	2	20%	5	50%	10	100%	2,50 (1,25/3,00)	
G3	1	10%	1	10%	1	10%	7	70%	10	100%	3,00 (2,25/3,00)	0,190¹

f: absolute frequency; n: sample size; IQR*: First Interquatil Range; IQR**: Third Interquatil Range; ¹Kruskal-Wallis test

## Data Availability

The datasets used and/or analyzed during the current study are available from the corresponding author.
